# Inter-Rater Variability of Prostate Lesion Segmentation on Multiparametric Prostate MRI

**DOI:** 10.3390/biomedicines11123309

**Published:** 2023-12-14

**Authors:** Thibaut Jeganathan, Emile Salgues, Ulrike Schick, Valentin Tissot, Georges Fournier, Antoine Valéri, Truong-An Nguyen, Vincent Bourbonne

**Affiliations:** 1 Radiology Department, University Hospital, 29200 Brest, France; thibaut.jeganathan@ch-cornouaille.fr (T.J.); emile.salgues@chu-brest.fr (E.S.); valentin.tissot@chu-brest.fr (V.T.); 2 Radiation Oncology Department, University Hospital, 29200 Brest, France; ulrike.schick@chu-brest.fr; 3 INSERM, LaTIM UMR 1101, University of Western Brittany, 29238 Brest, France; georges.fournier@chu-brest.fr (G.F.); antoine.valeri@chu-brest.fr (A.V.); truongan.nguyen@chu-brest.fr (T.-A.N.); 4 Urology Department, University Hospital, 29200 Brest, France

**Keywords:** prostate cancer, multiparametric MRI, segmentation, variability

## Abstract

Introduction: External radiotherapy is a major treatment for localized prostate cancer (PCa). Dose escalation to the whole prostate gland increases biochemical relapse-free survival but also acute and late toxicities. Dose escalation to the dominant index lesion (DIL) only is of growing interest. It requires a robust delineation of the DIL. In this context, we aimed to evaluate the inter-observer variability of DIL delineation. Material and Methods: Two junior radiologists and a senior radiation oncologist delineated DILs on 64 mpMRIs of patients with histologically confirmed PCa. For each mpMRI and each reader, eight individual DIL segmentations were delineated. These delineations were blindly performed from one another and resulted from the individual analysis of the T2, apparent diffusion coefficient (ADC), b2000, and dynamic contrast enhanced (DCE) sequences, as well as the analysis of combined sequences (T2ADC, T2ADCb2000, T2ADCDCE, and T2ADCb2000DCE). Delineation variability was assessed using the DICE coefficient, Jaccard index, Hausdorff distance measure, and mean distance to agreement. Results: T2, ADC, T2ADC, b2000, T2 + ADC + b2000, T2 + ADC + DCE, and T2 + ADC + b2000 + DCE sequences obtained DICE coefficients of 0.51, 0.50, 0.54, 0.52, 0.54, 0.55, 0.53, respectively, which are significantly higher than the perfusion sequence alone (0.35, *p* < 0.001). The analysis of other similarity metrics lead to similar results. The tumor volume and PI-RADS classification were positively correlated with the DICE scores. Conclusion: Our study showed that the contours of prostatic lesions were more reproducible on certain sequences but confirmed the great variability of prostatic contours with a maximum DICE coefficient calculated at 0.55 (joint analysis of T2, ADC, and perfusion sequences).

## 1. Introduction

With 1,400,000 new cases in 2020, prostate cancer (PCa) is the second most common cancer among men in the world, comprising 13.5% of all male cancer diagnoses (after lung cancer, which accounts for 14.5%) [1,2]. External beam radiation therapy (EBRT) is a major curative option for men with localized PCa [3]. Several dose-escalation studies have reported improved biochemical progression-free survival [4,5], with dose escalation being delivered to either the whole prostate gland or the dominant index lesion (DIL) itself.

The ASCENDE-RT trial showed that biochemical disease-free survival (bDFS) was increased in patients who received a brachytherapy boost to the whole prostate gland [6]. Given the rise in acute and late toxicities as a result of dose escalation to the whole prostate gland, there is a growing interest in more focused therapeutic approaches. In the FLAME trial, the addition of a focal boost to the DIL increased bDFS without affecting toxicity or quality of life. Patients with intermediate- or high-risk localized PCa were randomly assigned to the standard treatment arm (77 Gy in 35 fractions of 2,2 Gy) or the focal boost arm (focal boost up to 95 Gy to the macroscopic tumor in addition to the standard treatment). It has shown that the bDFS was significantly higher in the focal boost arm compared with the standard treatment arm (hazard ratio 0.45, 95% CI, 0.28 to 0.71, *p* < 0.001) with an acceptable toxicity profile [7]. Furthermore, in selected individuals with low to intermediate-risk PCa, focused brachytherapy with a high dose rate might obtain a similar relapse-free survival rate as the traditional whole-prostate brachytherapy, with less toxicity [8].

These treatments and clinical settings all have in common the need for a precise delineation of the DIL. Indeed, the delineation of the DIL has been identified as one of the main challenges preventing dose escalation to be broadly used [9]. 

Because of its high accuracy in lesion localization, multiparametric magnetic resonance imaging (mpMRI) is regarded as the current standard for detection [10,11,12,13] and biopsy planning [14,15]. The analysis of prostate mpMRIs requires dedicated training. Studies have previously focused on tumor volume estimation [16], but only a few studies have been conducted on the inter-reader variability of the delineation. With a cohort of 44 participants segmenting 20 prostate cases, the median DICE score was as low as 0.49, with an interquartile range (IQR) of 0.19–0.64 [17]. Furthermore, none of these studies has evaluated the inter-sequence variability. To this day, there is currently no consensus on the optimal magnetic resonance imaging (MRI) series (T2, apparent diffusion coefficient (ADC) cartography, diffusion-weighted imaging (DWI), or dynamic contrast-enhanced (DCE) MRI) to use for the delineation of the DIL. The robustness of the delineation itself was not previously evaluated and is not part of the European Society for Urogenital Radiology (ESUR) guidelines [18]. In this study, we thus aimed to evaluate the inter-reader variability of DIL delineation using mpMRIs. 

## 2. Materials and Methods

### 2.1. Population

A retrospective cohort of 64 patients with histologically confirmed localized PCa who did not previously undergo any treatment related to the PCa itself and who were treated at the Brest University Hospital, France, was used for the present study. A multiparametric prostate MRI was mandatory for all patients. No exclusion criteria regarding the prostate or the index lesion volume were used. Antiangiogenic medications, including 5-alpha reductase inhibitors, were not considered as contraindications. This study was approved by the Ethical Committee of the University Hospital Brest and registered on Clinical Trials as NCT05996289. All patients were sent a non-opposition form.

### 2.2. MRI Scan

The mpMRIs were performed following the ESUR and Prostate Imaging Reporting and Data System (PI-RADS) guidelines in use at the time of acquisition [18,19], using the Philips ACHIEVA 3T MR machine of University Hospital Brest (Philips Healthcare^©^, Amsterdam, The Netherlands) from 19 December 2011 to 25 March 2019.

The mpMRI protocol included the T2w turbo spin echo (TSE) DW-MRI with apparent diffusion coefficient (ADC) maps and dynamic contrast material-enhanced (DCE) sequences. We obtained DWI with calculated b0, b600, and b1000 sequences as well as an independent DWI b2000 sequence. The MRI parameters are summarized in Supplementary Table S1.

The DCE sequence was a T1 weighed sequence performed before, during, and after a bolus injection of a contrast agent (gadolinium) with a 5 s temporal resolution. An endorectal coil was not used.

To ensure comparability, each sequence was registered to the T2 sequence before segmentation using a 6-axis rigid registration. The registration was checked and validated by all readers, focusing on the prostate. The same registration was used for all readers.

### 2.3. Delineation of the Dominant Index Lesion (DIL)

The DILs were delineated by two junior diagnostic radiologists (three and four years of experience, respectively) and a senior radiation oncologist between 1 June 2021 and 10 July 2022. The two junior radiologists each had a 1-year fellowship in abdominal imaging (~250 MRI scans) while the senior radiation oncologist had 5 years of experience in prostate image analysis (~700 MRI scans).

The DIL was defined according to the PI-RADS classification [19]. All mpMRIs and PI-RADS classifications were reviewed according to the PI-RADS v2.1 guidelines [19] by a single radiologist with 15 years of experience in urology imaging.

A delineation workflow was specifically designed using the Workflow tool embedded in MIM Maestro software (MIM v7.1.2, Cleveland, OH, USA). The first step consisted in the registration of all sequences, using the T2 sequence as the reference, as previously described. Before contouring the DIL, each reader first contoured the prostate using the T2 sequence, and then the peripherical (PZ) and transitional zones (TZ). 

For each reader, eight DIL delineations were then realized. These delineations were blindly performed from one another and resulted from the individual analysis of the T2, apparent diffusion coefficient (ADC), b2000, and dynamic contrast enhanced (DCE) sequences, as well as the analysis of combined sequences (T2ADC, T2ADCb2000, T2ADCDCE, and the all-combined T2ADCb2000DCE sequence). Delineations were performed in the following order (T2, ADC, T2ADC, b2000, T2ADCb2000, DCE, T2ADCDCE, and the all-combined sequence) as shown in Supplementary Table S2. It has to be noted that each segmentation was masked before displaying the new sequence to be delineated. For instance, after delineating the DIL using the T2 sequence, both the T2 segmentation and T2 sequence were blinded and the ADC sequence was brought up, allowing the reader to delineate the DIL on the ADC sequence, blinded from the other sequences. A total of 4 delineations were added consisting in the union of T2 and ADC delineations (T2 + ADC), T2, ADC, and b2000 delineations (T2 + ADC + b2000), T2, ADC, and DCE delineations (T2 + ADC + DCE) and T2, ADC, b2000, and DCE delineations (T2 + ADC + b2000 + DCE).

It is noteworthy that due to the possible bias of the mis-selection of the DCE phases, the DCE sequence was analyzed using a semi-quantitative descriptive index, the Initial Area Under Gadolinium Curve (IAUCG) [20]. 

Each reader had the freedom to define and/or modify the viewing parameters, including the color table for the DCE analysis.

### 2.4. Similarity and Statistical Analysis

The spatial correlation between the different prostate contours and prostate tumoral lesion contours were evaluated using the following four geometric metrics: the DICE similarity coefficient (DSC), Jaccard index, Hausdorff distance (HD) measure, and mean distance to agreement (MDA). In order to evaluate the reproducibility of the contours between the different evaluators, an average of these variables was calculated for each sequence.

Similarity metrics were compared using the Student’s *t* test. A *p* value < 0.05 was considered as statistically significant.

As an explanatory analysis, the correlation between the similarity metrics and the features of age, prostate and lesion volume, as well as PI-RADS grading, was analyzed using the Spearman correlation coefficient. Based on this univariate analysis, statistically significant features were further processed through a multivariate logistic regression analysis.

Regarding the PI-RADS grading, a Student’s *t* test was used to compare the means of the similarity metrics between PI-RADS < 5 and PI-RADS = 5 patients.

### 2.5. Number of Patients

With the assumption that mpMRI would result in a 0.05 DSC gain, reaching 0.55 vs. 0.50 when compared to biparametric MRI (standard deviation of 0.08), a cohort of 64 patients will provide an 80% power with a type-1 error of 0.05.

## 3. Results

### 3.1. Population

Sixty-four patients were randomly selected among the overall cohort. Overall, the median age was 69 years (range 46–90). The PI-RADS classification was distributed as follows: 7 PI-RADS 3 lesions, 12 PI-RADS 4 lesions, and 45 PI-RADS 5 lesions. The characteristics of the patients and tumors are described in Table 1.

### 3.2. Similarity Analysis: Prostate, PZ, and TZ

The prostate volume appeared as similar with respective means of 41.25 mL, 43.77 mL, and 40.07 mL for T.J., E.S., and V.B., respectively (Supplementary Table S3). No significant difference was found between each reader except for the PZ delineation between ES and VB.

A high overlap between each prostate delineation with a mean DSC calculated at 0.89 (TJ/ES), 0.89 (TJ/VB), and 0.90 (ES/VB) was found (Table 2). It is noteworthy that the TZ and PZ delineations were associated with a higher variability with mean DSC values of 0.77 (TJ/ES), 0.79 (TJ/VB), and 0.78 (ES/VB) for the TZ delineations and 0.72 (TJ/ES), 0.75 (TJ/VB), and 0.72 (ES/VB) for the PZ delineations.

### 3.3. Similarity Analysis: Index Lesion

**Figure 1:**
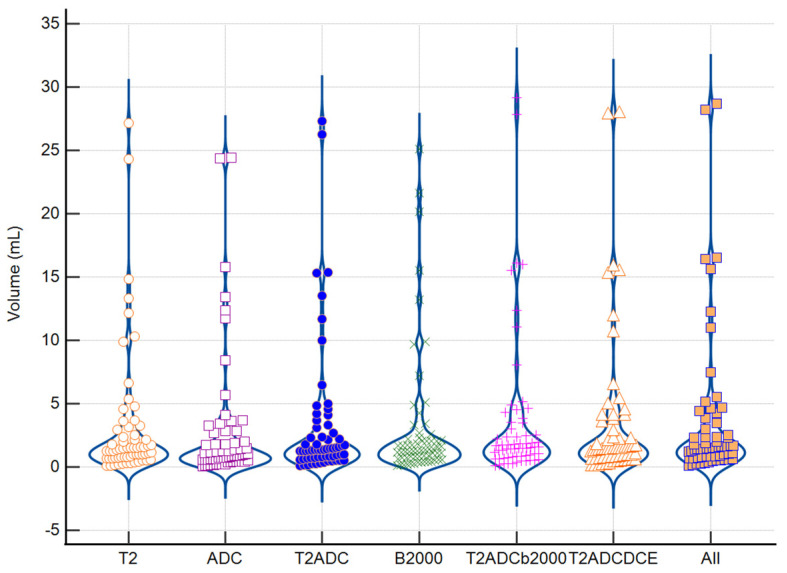
Mean of tumor volume delineated for each reader and each sequence. Abbreviations: ADC: Apparent Diffusion Coefficient, DCE: Dynamic Contrast Enhanced.

After contouring the prostate, PZ and TZ, readers focused on the prostate lesions whose averages volumes are shown in Figure 1 and supplementary Table S4. Displaying all sequences (T2, ADC, b2000 and DCE), the average delineated volume was 3.6 mL. As expected, the combined sequences had higher volumes than single sequences. Without taking the combined sequences into account, the lowest mean volumes were obtained using the DCE sequence (2.7 mL) and ADC map (2.8 mL), and the highest mean volume was obtained using the T2ADCb2000 (3.6 mL) and T2ADCb2000DCE (3.6 mL) sequences.

**Figure 2:**
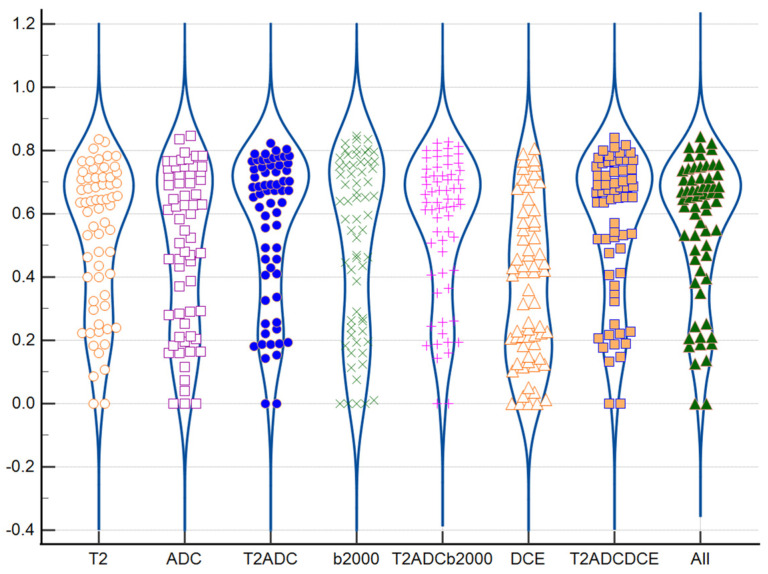
Inter-reader variability for the definition of the index lesion for each sequence (DICE). Abbreviations: DSC: DICE Similarity Coefficient, ADC: Apparent Diffusion Coefficient, DCE: Dynamic Contrast Enhanced.

Figure 2 as well as Supplementary Table S5 show the mean of the DSC coefficients obtained for each sequence and for each pair of readers. A notable variability between all DILs was found; the mean of the DSC was around 0.50 and 0.54, and even decreased to 0.35 for the DCE sequence. The T2, ADC, T2ADC, b2000, T2ADCb2000, T2ADCDCE and all-combined sequences obtained a significantly higher similarity than the DCE sequence (Table 3, *p* < 0.001).

We also noticed that the combined T2ADC and T2ADCb2000 sequences, with DSCs of 0.54 and 0.54, respectively, obtained higher DSCs than the single T2, ADC, and b2000 sequences (0.51, 0.50, and 0.52, respectively). Finally, the T2ADC and T2ADCb2000 sequences had significatively higher DSCs than ADCs (*p* = 0.0275 and *p* = 0.0261, respectively).

The delineations from the T2 + ADC, T2 + ADC + b2000, T2 + ADC + DCE, T2 + ADC + b2000 + DCE sequences obtained equal or higher DSCs than the single delineations from the T2ADC, T2ADCb2000, T2ADCDCE, and all-combined sequences. 

**Figure 3:**
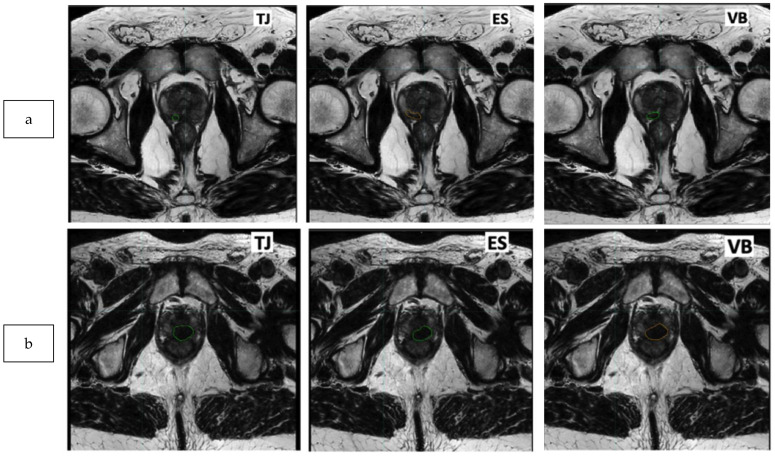
Example of tumor delineation on T2 sequence with low and high reproducibility ((**a**) mean of DICE: 0.31; (**b**) mean of DICE: 0.75).

Two patients (high variability and low variability) are presented in Figure 3a and Figure 3b, respectively.

The Jaccard index, HD measure, and MDA were consistent with these findings. The T2ADCDCE analysis obtained higher Jaccard index (0.42), and lower MDA (4.27 mm) values than other sequences (Supplementary Tables S6–S8), while the b2000 sequence scored the lowest mean Hausdorff distance score (10.35 mm).

We also evaluated the similarity of the contours between each sequence for each patient and each reader. The mean DSCs are shown in Supplementary Table S9. Table S9 shows that there was a high correlation between each sequence that contained a T2 sequence. However, a lower similarity was found between sequences that contained a T2 sequence and ADC, b2000, and DCE sequences. This could mean that the ADC, b2000 sequence, and DCE sequence presented different information for the tumor segmentation.

### 3.4. Impact of Tumoral Volume and PI-RADS Classification

A significant correlation between the tumor volume and the reproducibility was found for each sequence, with a positive correlation between the DSC and the lesion volume, as shown in Supplementary Table S10. A significant higher reproducibility was observed between the contours of the PI-RADS 5 lesions than others on each sequence (except the DCE sequence), as shown in Supplementary Table S11. The multivariate logistic regression analysis showed that only the PI-RADS classification remains as a significantly correlated feature.

## 4. Discussion

The aim of this study was to assess the variability between readers using different MRI sequences. Even if some sequences stood out from the others, we observed a high variability between the contours of the tumor lesions with a maximum mean DSC of 0.55 reached using the combined analysis of the T2, ADC, and DCE sequences. The combined T2 + ADC + DWI + DCE sequence reached the highest DSC (0.58), with the interpretation of these results being limited by the correlation between the DSC and the tumor volume itself. The higher DSC could be due to a larger volume as a result of the union of these contours. Few data are available regarding the inter-reader variability of prostate cancer segmentation. With sixteen participants (four radiologists, four urologists, four urology trainees, and four non-clinicians), a high variance was found in the segmentation of two prostate tumors using single-slice images (mean DSC of 0.81 and 0.58) [21]. Van Schie et al. demonstrated that there were inter-institutional variations in the interpretation of mpMRIs (T2, ADC, and DCE-MRI-Derived Volume Transfer Constant (K trans)) for the prostate cancer delineation of 260 patients in the FLAME study cohort [22]. Similarity indexes were not available. 

In a previous study focusing on tumor volume estimation only, DCE sequences achieved the highest result in comparison to the histopathological ground truth. It is noteworthy that, despite reaching the highest correlation, DCE sequences still lead to a substantial underestimation of the DIL volume [16], and suffered from a low reproducibility in our study. It is also noteworthy that, in our study, DCE delineation relied on a parametric map and not the DCE phases themselves. Each DCE sequence is comprised of 30 to 50 phases. Choosing a reference phase for the delineation would have exposed the risk that each reader selects a different phase. The delineation would have been possibly biased by the selection of the phase more than by the sequence itself. We thus used a semi-quantitative descriptive index, the IAUCG [20].

A precise tumor delineation is essential for the development and evaluation of focal treatments in prostate cancer. Our results highlight the high variability between three readers. It would be beneficial to train the observers to improve the consensus of delineations using mpMRIs and/or apply a margin to the delineated tumor to include more tumor tissue. It is noteworthy that in the FLAME trial [7], researchers did not apply a margin to the delineated tumor. With a mean MDA of 4.3 for the T2ADC delineation, a 5 mm margin could be proposed to account for inter-reader variability. Training and/or using additional margins are two solutions. Other efforts have been made to improve delineation reproducibility. The ReIGNITE study evaluated the impact of restriction spectrum imaging restriction scores on inter-reader variability, successfully increasing the median DSC from 0.48 (IQR 0.19–0.64) to 0.66 (IQR 0.55–0.73). Automatic segmentation tools for DIL delineation could possibly outperform inter-reader variability. While automatic prostate segmentation is often highly reproducible and robust [23,24], the automatic segmentation of the DIL performs poorly. Focusing on low-risk prostate cancer patients and with T2, B800, and ADC maps as input data, a model reached AUCs of 0.65, 0.73, and 0.89 for volumes >0.03 cc, >0.1 cc, and >0.5 cc, respectively. Specificity percentages were as low as 43%, 52%, and 74%, depending on the volume cut-off point. DSCs were not available [25]. With the addition of transfer learning and test-time augmentation, a model using a convolutional neural network approach reached a DSC of 0.59 using histopathology as the gold standard [26]. Tsui et al. evaluated the impact of an automatically generated DIL, leading to the conclusion that dosimetry planning was significantly impacted by the accuracy of the DIL delineation [27]. Finally, other imaging modalities, such as PSMA-positron emission tomography (PET), could be used to segment the DIL [28]. The impact of the choice of window levelling should be studied [29]. 

Despite their interesting character, these results highlight the difficulty in prostate cancer segmentation, with the only comparison being either to the histopathology data and its inherent limitation (fusion, cancer retraction after staining), or to a single expert segmentation. The choice of the similarity metric (DSC) can be challenged. While the DSC reflects the extent of the overlap [30], other metrics could be adapted to the delineation task, such as the hit criteria used in the PI-CAI challenge (minimum overlap between the detection and the ground truth) [31]. 

Despite our efforts in the design of the present study, several limitations should be acknowledged. The intra-reader variability was not evaluated. Our cohort focuses on a single scanner evaluation and on patients who were further treated either with surgery or EBRT. By definition, this leads to a selection bias, limiting the external validity of our results in a PI-RADS < 5 cohort. However, it is interesting to note that despite a cohort of >70% PI-RADS 5 DILs, the highest DSC was as low as 0.55. This low DICE score signifies the need to exercise caution for margin-free dose-escalation approaches and will be compared to expert-only results in an additional study. The DCE sequences were analyzed using the IAUCG and lead to an unexpected high variability and smaller mean volumes in comparison to T2 and ADC sequences. While the IAUCG eased the delineation workflow, it is possible that the use of the IAUCG was sub-optimal. It is noteworthy that the use of DCE sequences is controversial [32,33,34] and biparametric MRIs (T2 and ADC) are more available than mpMRIs, considering the long acquisition time of DCE MRIs, and the potential risks and additional costs of gadolinium injections.

This cohort will be used to develop an automatic segmentation algorithm. Indeed, we believe that the variability of prostate cancer segmentation should be taken into account when designing and building automatic segmentation tools. Complementarity between MRI sequences should also be incorporated. Multiparametric MRIs could thus be used to its full potential by combining the input on differential segmentations depending on the sequence. Furthers studies could focus on the factors influencing the variability of prostate cancer segmentation and evaluate its dosimetric impact.

## 5. Conclusions

A high variability in prostate cancer delineation was observed, with T2, T2ADC, b2000, T2ADCb2000, T2ADCDCE, and all-combined sequences reaching the highest, yet very moderate, similarity scores. This inter-reader variability should be taken into account when planning focal treatments, such as stereotactic radiotherapy or dose-escalated radiotherapy, but also when designing automatic segmentation tools. 

## Figures and Tables

**Table 1:** Patients’ characteristics.

Characteristics of Patients	Number of Patients = 64
Age (median (range))	69 (46–90)
PI-RADS Classification	
PI-RADS 3	7/64 (10.9)
PI-RADS 4	12/64 (18.8)
PI-RADS 5	45/64 (70.3)
Mean PSA (ng/mL)	9.1
PSA range (ng/mL)	
<10	41/64 (64.1%)
10–20	20/64 (31.3%)
>20	3/64 (4.7%)
ISUP Class	
5	9/64 (14/1%)
4	9/64 (14/1%)
3	9/64 (14/1%)
2	19/64 (29.7%)
1	18/64 (28.1%)

Abbreviations: PSA: Prostate Specific Antigen, ISUP: International Society of Urological Pathology.

**Table 2:** Inter-reader variability of prostate, transitional, and peripherical zone delineations.

		Prostate		Transitional Zone		Peripherical Zone	
Pair of Readers	Variable	Mean	SD	Mean	SD	Mean	SD
TJ/ES	DICE	0.89	0.03	0.77	0.13	0.72	0.12
TJ/VB	DICE	0.89	0.03	0.79	0.11	0.75	0.06
VB/ES	DICE	0.90	0.04	0.78	0.11	0.72	0.12
All	DICE	0.90	0.03	0.77	0.13	0.72	0.10

Abbreviation: SD: standard deviation.

**Table 3:** Difference in mean DICE scores between each sequence (*p*-values).

	T2	ADC	T2ADC	b2000	T2ADCb2000	DCE	T2ADCDCE	All	T2 + ADC	T2 + ADC + b2000	T2 + ADC + DCE	T2 + ADC + b2000 + DCE
T2	1.00	0.27	0.25	0.73	0.25	<0.0001 ^†^	0.23	0.27	0.15	0.15	0.15	0.02 ^†^
ADC	0.27	1.00	0.03 ^†^	0.49	0.03 ^†^	0.01 ^†^	0.02 ^†^	0.03 ^†^	0.01 ^†^	0.01 ^†^	0.01 ^†^	0.01 ^†^
T2ADC	0.25	0.03 ^†^	1.00	0.16	1.00	<0.0001 ^†^	0.95	0.90	0.77	0.77	0.80	0.28
B2000	0.73	0.49	0.16	1.00	0.16	<0.0001 ^†^	0.14	0.19	0.09	0.09	0.10	0.02 ^†^
T2ADCb2000	0.25	0.03 ^†^	1.00	0.16	1.00	<0.0001 ^†^	0.95	0.90	0.77	0.77	0.80	0.27
DCE	<0.0001 ^†^	0.00 ^†^	<0.0001 ^†^	<0.0001 ^†^	<0.0001 ^†^	1.00	<0.0001 ^†^	<0.0001 ^†^	<0.0001 ^†^	<0.0001 ^†^	<0.0001 ^†^	<0.0001 ^†^
T2ADCDCE	0.23	0.02 ^†^	0.95	0.14	0.95	<0.0001 ^†^	1.00	0.85	0.81	0.81	0.84	0.30
All	0.27	0.03 ^†^	0.90	0.19	0.90	<0.0001 ^†^	0.85	1.00	0.67	0.67	0.70	0.22
T2 + ADC	0.15	0.01 ^†^	0.77	0.09	0.77	<0.0001 ^†^	0.81	0.67	1.00	1.00	0.96	0.43
T2 + ADC + b2000	0.15	0.01 ^†^	0.77	0.09	0.77	<0.0001 ^†^	0.81	0.67	1.00	1.00	0.96	0.43
T2 + ADC + DCE	0.15	0.01 ^†^	0.80	0.10	0.80	<0.0001 ^†^	0.84	0.70	0.96	0.96	1.00	0.40
T2 + ADC + b2000 + DCE	0.02 ^†^	0.00 ^†^	0.28	0.02 ^†^	0.27	<0.0001 ^†^	0.30	0.22	0.43	0.43	0.40	1.00

Abbreviations: SD: standard deviation, ADC: Apparent Diffusion Coefficient, DCE: Dynamic Contrast Enhanced, ^†^: *p* < 0.05.

## Data Availability

Data are available upon request to the corresponding author.

## Supplementary Materials

The following supporting information can be downloaded at: https://www.mdpi.com/article/10.3390/biomedicines11123309/s1, Table S1: MRI parameters; Table S2: Delineation workflow example; Table S3: Prostate, transitional, and peripherical zone delineation (mean); Table S4: Mean of tumor volume contoured for each reader and each sequence; Table S5: Inter-reader variability for the definition of the index lesion for each sequence (DICE); Table S6: Inter-reader variability for the definition of the index lesion for each sequence (Jaccard); Table S7: Inter-reader variability for the definition of the index lesion for each sequence (Max Hausdorff); Table S8: Inter-reader variability for the definition of the index lesion for each sequence (Mean distance to agreement); Table S9: Intersequence contour similarity evaluation (mean DICE coefficients); Table S10: Impact of tumor volume and PI-RADS on inter-reader variability; Table S11: Comparison between mean DICE coefficients for each sequence.
