# Supplementary material for: Inter-Rater Variability of Prostate Lesion Segmentation on Multiparametric Prostate MRI
Source: Biomedicines. 2023 Dec 14;11(12):3309. doi: 10.3390/biomedicines11123309 (PMC10741937; doi:10.3390/biomedicines11123309)
Supplement: Supplementary file 1 [file biomedicines-11-03309-s001.zip › biomedicines-2766288-supplementary.pdf]

Supplementary Materials

Supplementary Table S1: MRI parameters

Supplementary Table S2: Delineation workflow example

Supplementary Table S3: Prostate, transitional and peripherical zones delineation (mean)

Supplementary Table S4: Mean of tumor volume delineated for each reader and each sequence.

Supplementary Table S5: Inter-reader variability for the definition of the index lesion for each sequence (Dice).

Supplementary Table S6: Inter-reader variability for the definition of the index lesion for each sequence (Jaccard)

Supplementary Table S7: Inter-reader variability for the definition of the index lesion for each sequence (Max Hausdorff)

Supplementary Table S8: Inter-reader variability for the definition of the index lesion for each sequence (Mean distance to agreement)

Supplementary Table S9: Intersequence contours similarity evaluation (mean dice coefficients)

Supplementary Table S10: Impact of tumor volume and PI-RADS on inter-reader variability

Supplementary Table S11: Comparison between mean dice coefficients for each sequence

Supplementary Table S1: MRI parameters

Sequence	TR (ms) (mean, range)	TE (ms) (mean, range)	Slice thickness (mm) (median, range)	Matrix (range)	FOV (mm, range)	Pixel size (mm, range)	Total scan time (sec) (mean, range)
**T2 TSE**	4290.3 (3845.4	90.0 (90.0 – 90.0)	3.0 (3.0 – 3.0)	256 x 215 –	180 x 180 –	0.35 x 0.35	186.5 (146.1 –
**Diffusion**	3768.6 (1800.0	87.1 (77.9 – 100.5)	3.7 (3.0 – 5.0)	64 x 61 - 116 x	140x140 – 373 x	0.97 x 0.97 – 1.46 x	181.2 (147.6 – 251.1)
**DCE**	3.1 (3.0 – 3.2)	1.5 (1.4 – 1.5)	3.0 (3.0 – 3.0)	120 x 120	180x180	1.02 x 1.02	234.3 (164.8 – 302.1)

Supplementary Table S2: Delineation workflow example

**Step 1.** Delineation of prostate contour	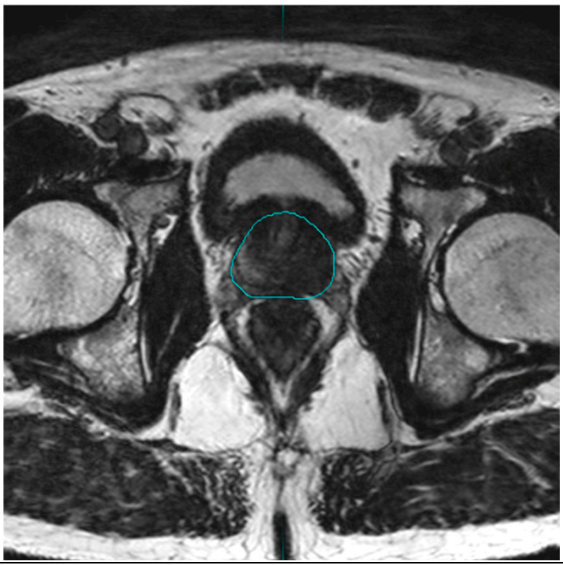
**Step 2.** Delineation of peripherical zone contour	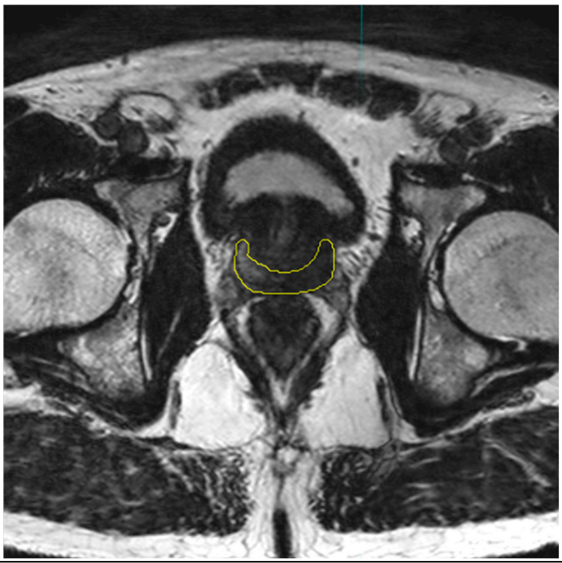

**Step 3.** Delineation of transitional zone contour	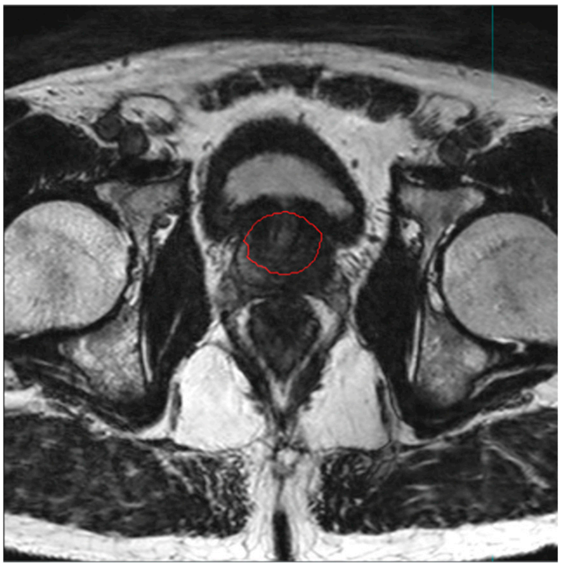
**Step 4.** Delineation of tumor lesion on T2	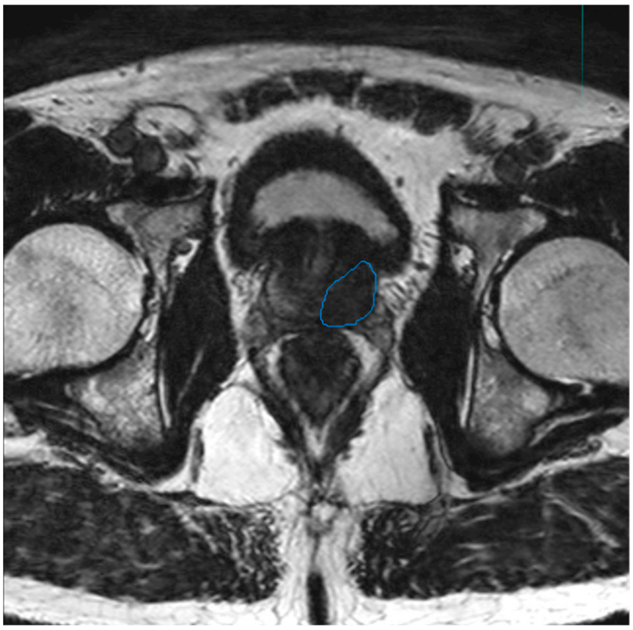
**Step 5.** Delineation of tumor lesion on ADC	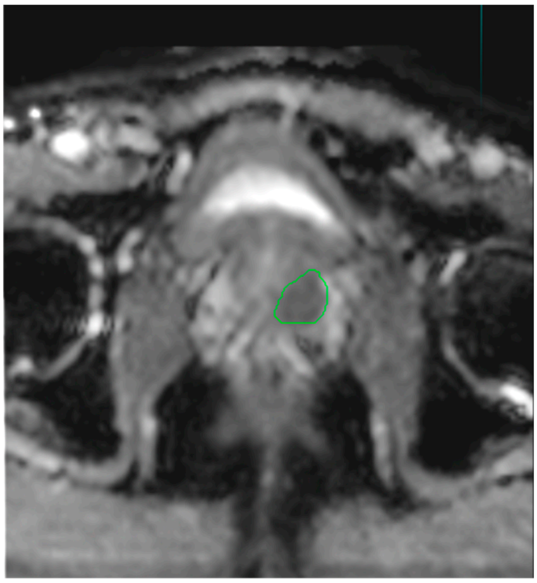

**Step 6.** Delineation of tumor lesion with T2 and ADC	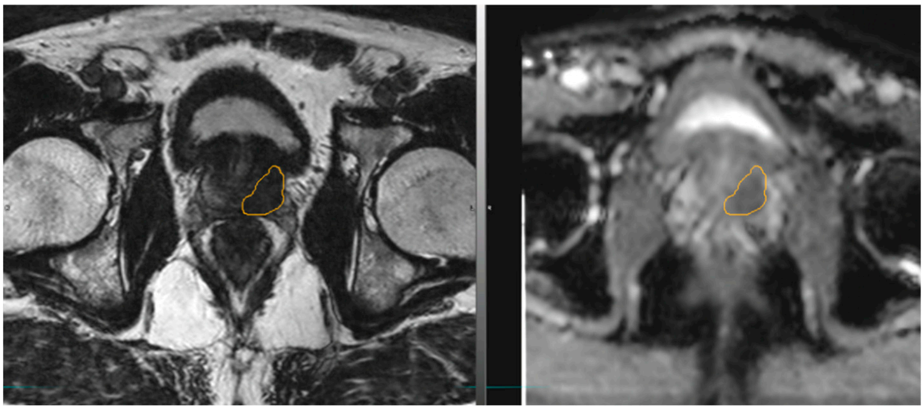
**Step 7.** Delineation of tumor lesion on DWI (b2000)	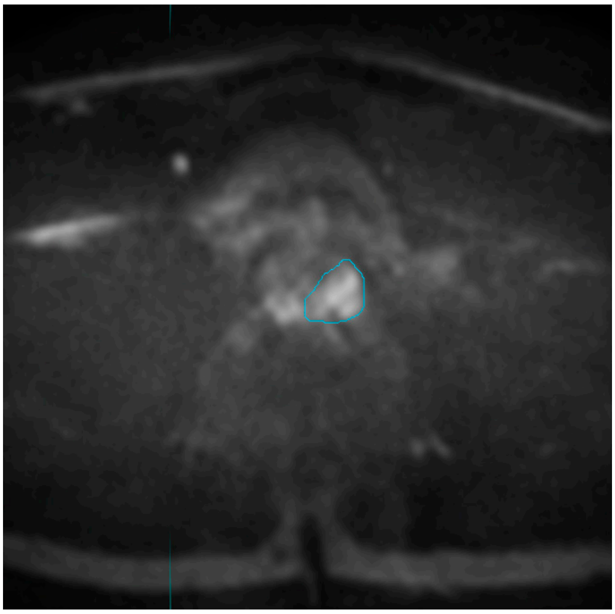
**Step 8.** Delineation of tumor lesion with T2, DWI (b2000) and ADC	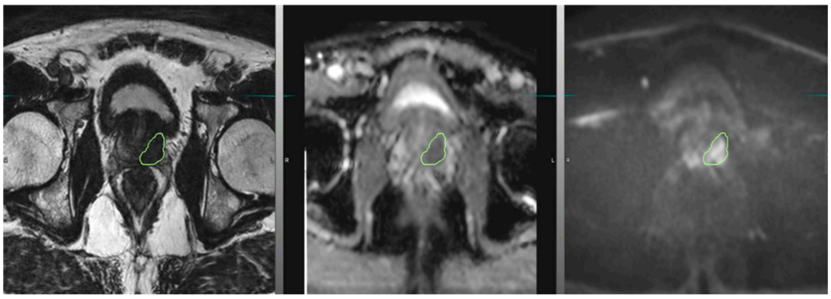

**Step 9.** Delineation of tumor lesion on DCE (choice of color tables at the discretion of the reader)	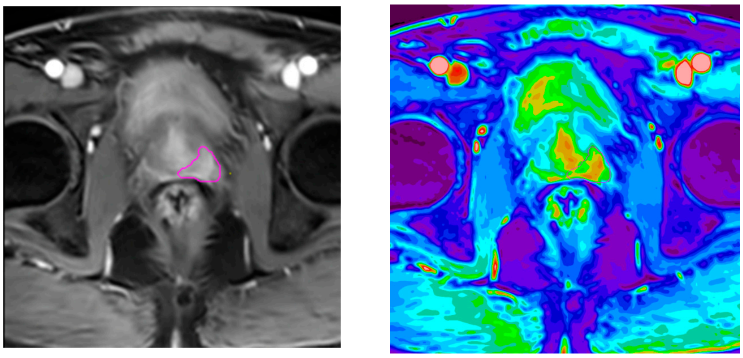
**Step 10.** Delineation of tumor lesion with T2, ADC and DCE	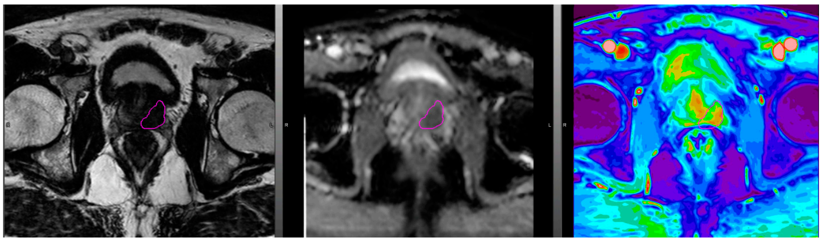

**Step 11.** Delineation of tumor with T2, ADC, Dwi (b2000) and DCE (All-combined)	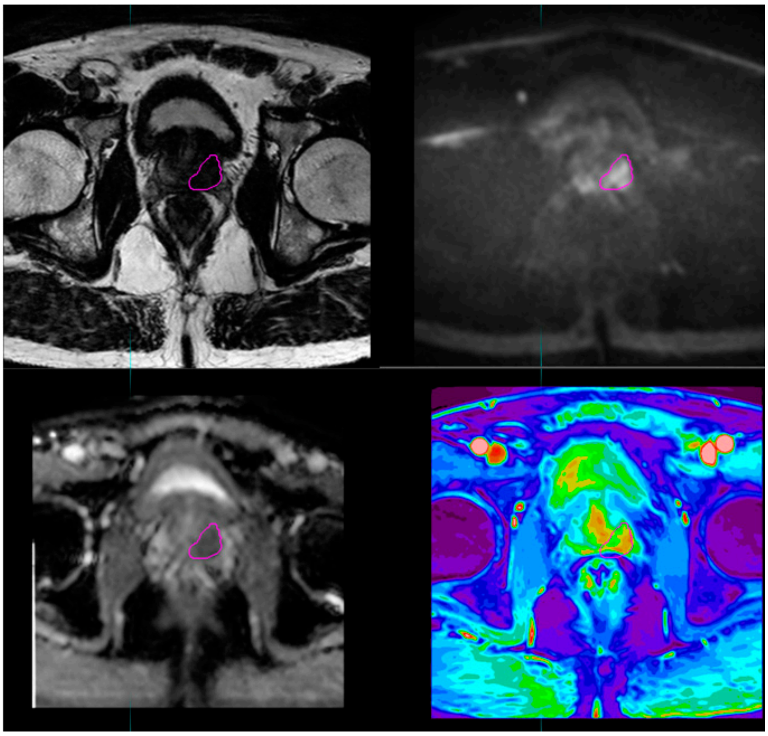
**Step 12.** Summary of all contours with the four added contours (T2+ADC, T2+ADC+ b2000, T2+ADC+ DCE, T2+ADC+ b2000+ DCE)	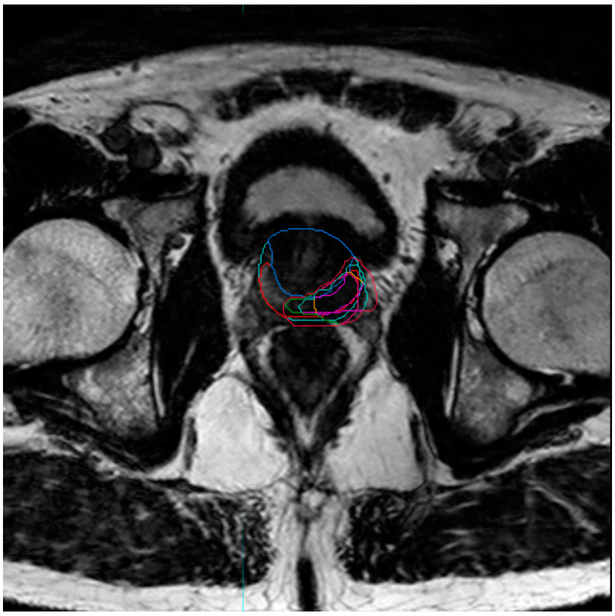

Supplementary Table S3: Prostate, transitional and peripherical zones delineation (mean volumes)

	Prostate				Transitional zone (ml)				Peripherical zone (ml)			
	Volume (mL)	SD	Statistical difference		Volume (ml)	SD	Statistical difference		Volume (mL)	SD	Statistical difference	
			Comparison	p			Comparison	p			Comparison	p
**TJ**	41.3	17.2	VB	0.41	24.2	12.9	VB	0.53	16.1	6.5	VB	0.07
			ES	0.70			ES	0.97			ES	0.34
**ES**	43.8	17.0	TJ	0.70	24.1	13.2	TJ	0.97	14.9	7.7	TJ	0.34
			VB	0.23			VB	0.51			VB	0.01
**VB**	40.1	17.5	TJ	0.41	25.7	13.9	TJ	0.53	18.2	6.7	TJ	0.07
			ES	0.23			ES	0.51			ES	0.01
**All**	41.7	17.2			24.7	13.3			16.4	7.1		

Abbreviation: SD: standard deviation

Supplementary Table S4: Mean of tumor volume delineated for each reader and each sequence.

	TJ		ES		VB		All
**Sequences**	Mean (ml)	SD	Mean (ml)	SD	Mean (ml)	SD	Mean (ml)
**T2**	2.81	4.7	4.0	6.8	2.67	4.5	3.2
**ADC**	3.18	5.6	3.3	6.4	2.0	3.7	2.8
**T2ADC**	3.04	5.2	4.0	6.8	2.9	4.9	3.3
**b2000**	3.8	6.2	3.6	6.3	2.3	4.0	3.2
**T2ADCb2000**	3.3	5.7	4.3	7.0	3.2	5.2	3.6
**DCE**	3.9	7.2	2.4	5.9	1.9	3.7	2.7
**T2ADCDCE**	3.2	5.4	4.1	7.0	3.1	5.2	3.5
**All-combined**	3.3	5.6	4.2	7.0	3.4	5.4	3.6
**T2+ADC**	4.0	6.3	4.7	7.9	3.1	5.2	3.9
**T2+ADC+b2000**	4.0	6.3	4.7	7.8	3.2	5.2	3.9
**T2+ADC+DCE**	5.3	8.2	5.2	9.0	3.6	5.7	4.7
**T2+ADC+b2000+DCE**	5.9	8.6	5.8	9.5	4.1	6.2	5.3

Abbreviations: SD: standard deviation, ADC: Apparent Diffusion Coefficient, DCE: Dynamic Contrast Enhanced.

Supplementary Table S5. Inter-reader variability for the definition of the index lesion for each sequence (Dice).

	TJ/ES		TJ/VB		ES/VB		All	
	**Mean**	**SD**	**Mean**	**SD**	**Mean**	**SD**	**Mean**	**SD**
**T2**	0.51	0.28	0.52	0.28	0.51	0.28	0.51	0.28
**ADC**	0.51	0.29	0.49	0.27	0.51	0.29	0.50	0.29
**T2ADC**	0.54	0.28	0.55	0.28	0.54	0.28	0.54	0.28
**b2000**	0.54	0.32	0.49	0.28	0.54	0.31	0.52	0.31
**T2ADCb2000**	0.54	0.27	0.54	0.28	0.54	0.27	0.54	0.27
**DCE**	0.32	0.28	0.39	0.28	0.32	0.28	0.35	0.28
**T2ADCDCE**	0.54	0.27	0.55	0.28	0.54	0.27	0.55	0.28
**All-combined**	0.53	0.27	0.54	0.27	0.53	0.27	0.53	0.27
**T2+ADC**	0.56	0.29	0.56	0.28	0.56	0.29	0.56	0.29
**T2+ADC+b2000**	0.56	0.29	0.56	0.28	0.56	0.29	0.56	0.29
**T2+ADC+DCE**	0.56	0.27	0.54	0.27	0.56	0.27	0.55	0.27
**T2+ADC+b2000+DCE**	0.58	0.27	0.58	0.26	0.58	0.27	0.58	0.27

Supplementary Table S6: Inter-reader variability for the definition of the index lesion for each sequence (Jaccard)

	TJ/ES		TJ/VB		ES/VB		All	
	**Mean**	**SD**	**Mean**	**SD**	**Mean**	**SD**	**Mean**	**SD**
**T2**	0.39	0.23	0.39	0.24	0.39	0.23	0.39	0.23
**ADC**	0.39	0.24	0.37	0.23	0.39	0.24	0.38	0.24
**T2ADC**	0.41	0.23	0.43	0.24	0.41	0.23	0.42	0.23
**b2000**	0.43	0.27	0.37	0.24	0.43	0.27	0.41	0.26
**T2ADCb2000**	0.41	0.23	0.42	0.23	0.41	0.23	0.41	0.23
**DCE**	0.23	0.22	0.28	0.23	0.23	0.22	0.25	0.22
**T2ADCDCE**	0.42	0.23	0.43	0.24	0.42	0.23	0.42	0.23
**All-combined**	0.40	0.22	0.41	0.22	0.40	0.22	0.41	0.22
**T2+ADC**	0.44	0.24	0.43	0.24	0.44	0.24	0.44	0.24
**T2+ADC+b2000**	0.43	0.24	0.43	0.24	0.43	0.24	0.43	0.24
**T2+ADC+DCE**	0.43	0.23	0.42	0.22	0.43	0.23	0.43	0.23
**T2+ADC+b2000+DCE**	0.46	0.23	0.44	0.22	0.46	0.23	0.45	0.23

Abbreviations: SD: standard deviation, ADC: Apparent Diffusion Coefficient, DCE: Dynamic Contrast Enhanced

Supplementary Table S7: Inter-reader variability for the definition of the index lesion for each sequence (Max Hausdorff)

	TJ/ES		TJ/VB		ES/VB		All	
	**Mean (mm)**	**SD**	**Mean (mm)**	**SD**	**Mean (mm)**	**SD**	**Mean (mm)**	**SD**
**T2**	0.39	0.23	0.39	0.24	0.39	0.23	11.07	9.08
**ADC**	0.39	0.24	0.37	0.23	0.39	0.24	10.93	9.24
**T2ADC**	0.41	0.23	0.43	0.24	0.41	0.23	10.52	9.10
**b2000**	0.43	0.27	0.37	0.24	0.43	0.27	10.35	9.43
**T2ADCb2000**	0.41	0.23	0.42	0.23	0.41	0.23	10.67	8.92
**DCE**	0.23	0.22	0.28	0.23	0.23	0.22	13.82	9.22
**T2ADCDCE**	0.42	0.23	0.43	0.24	0.42	0.23	10.37	8.95
**All-combined**	0.40	0.22	0.41	0.22	0.40	0.22	10.61	8.82
**T2+ADC**	0.44	0.24	0.43	0.24	0.44	0.24	10.99	9.07
**T2+ADC+b2000**	0.43	0.24	0.43	0.24	0.43	0.24	11.02	9.05
**T2+ADC+DCE**	0.43	0.23	0.42	0.22	0.43	0.23	11.45	8.87
**T2+ADC+b2000+DCE**	0.46	0.23	0.44	0.22	0.46	0.23	11.60	8.87

Abbreviations: SD: standard deviation, ADC: Apparent Diffusion Coefficient, DCE: Dynamic Contrast Enhanced

Supplementary Table S8: Inter-reader variability for the definition of the index lesion for each sequence (Mean distance to agreement)

	TJ/ES		TJ/VB		ES/VB		All	
	**Mean (mm)**	**SD**	**Mean (mm)**	**SD**	**Mean (mm)**	**SD**	**Mean (mm)**	**SD**
**T2**	4.81	7.87	4.03	6.49	4.81	7.87	4.55	7.41
**ADC**	4.86	7.73	4.25	6.67	4.86	7.73	4.66	7.37
**T2ADC**	4.56	7.88	3.82	6.58	4.56	7.88	4.31	7.45
**b2000**	4.76	7.73	4.32	6.51	4.76	7.73	4.61	7.32
**T2ADCb2000**	4.66	7.94	3.83	6.54	4.66	7.94	4.38	7.47
**DCE**	7.04	8.04	5.27	6.82	7.04	8.04	6.45	7.64
**T2ADCDCE**	4.53	7.91	3.76	6.48	4.53	7.91	4.27	7.43
**All-combined**	4.62	7.84	3.81	6.50	4.62	7.84	4.35	7.40
**T2+ADC**	4.54	7.65	3.78	6.27	4.54	7.65	4.29	7.19
**T2+ADC+b2000**	4.54	7.65	3.79	6.27	4.54	7.65	4.29	7.19
**T2+ADC+DCE**	4.65	7.57	3.85	5.98	4.65	7.57	4.38	7.04
**T2+ADC+b2000+DCE**	4.47	7.32	3.67	5.77	4.47	7.32	4.20	6.81

Abbreviations: SD: standard deviation, ADC: Apparent Diffusion Coefficient, DCE: Dynamic Contrast Enhanced

Supplementary Table S9: Intersequence contours similarity evaluation (mean dice coefficients)

Sequence	T2	ADC	T2ADC	b2000	T2ADC b2000	DCE	T2ADC DCE	All-combined
**T2**	**1.00**	0.55	0.91‡	0.48	0.86‡	0.42	0.88‡	0.86‡
**ADC**	0.55	**1.00**	0.59	0.50	0.57	0.40	0.58	0.57
**T2ADC**	0.91 ‡	0.59	**1.00**	0.50	0.92‡	0.44	0.95‡	0.92‡
**b2000**	0.48	0.50	0.50	**1.00**	0.56	0.42	0.51	0.54
**T2ADCb2000**	0.86 ‡	0.57	0.92‡	0.56	**1.00**	0.45	0.91‡	0.94‡
**DCE**	0.42	0.40	0.44	0.42	0.45	**1.00**	0.47	0.46
**T2ADCDCE**	0.88 ‡	0.58	0.95‡	0.51	0.91‡	0.47	**1.00**	0.94‡
**All-combined**	0.86 ‡	0.57	0.92‡	0.54	0.94‡	0.46	0.94‡	**1.00**

Abbreviations: SD: standard deviation, ADC: Apparent Diffusion Coefficient, DCE: Dynamic Contrast Enhanced, ‡ : DICE > 0.70

Supplementary Table S10: Impact of tumor volume and PI-RADS on inter-reader variability

	**Correlation to tumor volume**		**Correlation to PI-RADS classification**	
**Sequence**	**Spearman coefficient**	**p**	**Spearman coefficient**	**p**
**T2**	0.361	0.0011	0.38	0.0019
**ADC**	0.56	<0.0001	0.47	0.0001
**T2ADC**	0.483	0.0001	0.40	0.001
**b2000**	0.392	0.0014	0.44	0.0003
**T2ADCb2000**	0.58	<0.0001	0.43	0.0004
**DCE**	0.345	0.0053	0.21	0.0888
**T2ADCDCE**	0.529	<0.0001	0.46	0.0002
**All**	0.553	<0.0001	0.47	0.0001
**T2+ADC**	0.457	0.0001	0.47	0.0001
**T2+ADC+b2000**	0.461	0.0001	0.47	0.0001

**T2+ADC+DCE**	0.463	0.0001	0.42	0.0006
**T2+ADC+b2000+DCE**	0.482	0.0001	0.45	0.0002

Abbreviations: SD: standard deviation, ADC: Apparent Diffusion Coefficient, DCE: Dynamic Contrast Enhanced

Supplementary Table S11: Comparison between mean dice coefficients for each sequence

	**PI-RADS < 5**	**PI-RADS = 5**	**p**
	**Mean Dice**		
**T2**	0.37	0.60	0.0002
**ADC**	0.31	0.57	0.0001
**T2ADC**	0.40	0.63	0.0002
**b2000**	0.33	0.59	0.0003
**T2ADCb2000**	0.39	0.64	< 0.0001
**DCE**	0.30	0.40	0.1362
**T2ADCDCE**	0.38	0.64	< 0.0001
**All**	0.38	0.63	< 0.0001
**T2+ADC**	0.39	0.65	< 0.0001
**T2+ADC+b2000**	0.39	0.65	< 0.0001
**T2+ADC+DCE**	0.39	0.64	< 0.0001
**T2+ADC+b2000+DCE**	0.41	0.67	< 0.0001

Abbreviations: SD: standard deviation, ADC: Apparent Diffusion Coefficient, DCE: Dynamic Contrast Enhanced
